# Pivotal Enzyme in Glutamate Metabolism of Poly-γ-Glutamate-Producing Microbes

**DOI:** 10.3390/life3010181

**Published:** 2013-02-06

**Authors:** Makoto Ashiuchi, Takashi Yamamoto, Tohru Kamei

**Affiliations:** Graduate School of Integrated Arts and Sciences, Kochi University, Nankoku, Kochi 783-8502, Japan; E-Mails: b094m021@s.kochi-u.ac.jp (T.Y.); b094m173@s.kochi-u.ac.jp (T.K.)

**Keywords:** L-glutamate sources, glutamate racemase, PGA chirality, archaea, bacteria

## Abstract

The extremely halophilic archaeon *Natrialba aegyptiaca *secretes the L-homo type of poly-γ-glutamate (PGA) as an extremolyte. We examined the enzymes involved in glutamate metabolism and verified the presence of L-glutamate dehydrogenases, L-aspartate aminotransferase, and L-glutamate synthase. However, neither glutamate racemase nor D-amino acid aminotransferase activity was detected, suggesting the absence of sources of D-glutamate. In contrast, D-glutamate-rich PGA producers mostly possess such intracellular sources of D-glutamate. The results of our present study indicate that the D-glutamate-anabolic enzyme “glutamate racemase” is pivotal in the biosynthesis of PGA.

## 1. Introduction

**Figure 1:**
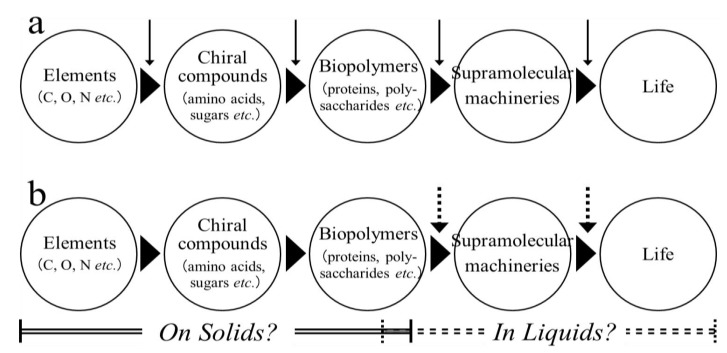
A speculative chemical process in which water made life possible**. **(a) A general scheme in which water continuously participated in all steps in the creation of life (*solid arrows*). (b) An alternative view indicating that water participated intermittently in some limited reactions (*dotted arrows*). When various materials containing homochiral polymers are assembled to generate supramolecular machineries with biological functions, several physicochemical forces mediated by water molecules, *e.g.*, the formation of hydrophobic interaction and hydrogen bonds, are indispensable. Essential water molecules may participate in a later stage of the process.

“No homochirality, no life” [1]. This saying may be accepted as true from the study of biologically essential polymers. Most proteins and polysaccharides are indeed comprised of L-amino acids and D-dominant sugars, respectively, and are considered homochiral molecules. Furthermore, D-ribose is required for *in vivo *synthesis of nucleic acids. Although a large body of evidence indicates that life originated in an aqueous environment and was accompanied by the generation of such chiral molecules of different sizes (Figure 1a), there are some contradictions from the perspective of polymer chemistry. Generally, the chemical polymerization of substrate monomers is performed under water-limited (or anhydrous) conditions, because this process depends on condensation reactions involving dehydration (removal of a H_2_O molecule) between two functional groups of interest. Biopolymers in water will therefore undergo hydrolysis (for degradation) rather than elongation (for synthesis). This stance does not contradict knowledge that *in vivo *syntheses of biopolymers involves strictly water-limited spaces, *e.g.*, the cell membranes or hydrophobic clefts of supramolecular machineries. Also to be considered is that the homochirality of substrate monomers facilitates cleavage catalyzed by water [2,3]. Maybe, water was indispensable for the final changes in the chemical transformations that made life possible (Figure 1b). It is well known that a crystal is formed by the systematic assembly of molecules possessing the same conformation. Thus, if researchers had proved that the primitive, spontaneous polymerization reactions occurred in the absence of water, specifically during crystallization, they may not be perplexed by the origin of homochirality in biomass. In contrast, the search for the occurrence of heterochirality in the biological world is becoming a rather attractive pursuit for researchers.

The extremely halophilic archaeon *Natrialba aegyptiaca* synthesizes the extracellular polyamide poly-γ-glutamate (L-PGA), which consists of more than 10,000 molecules of L-glutamate polymerized *via *γ-amide linkages and encompasses a chiral center in every glutamyl residue. This archaeal polymer therefore exhibits protein-like homochirality. However, well-characterized PGAs generally contain D-glutamate [4] and endogenous D-glutamate is likely to be a substrate for bacterial PGA synthetases [5]. In the present study, we focus on glutamate metabolism by *N. aegyptiaca* and also discuss a pivotal enzyme involved in the synthesis of microbial PGAs.

## 2. Results and Discussion

### 2.1. Determination of Sources of L-glutamate in the Production of L-PGA by N. aegyptiaca

**Figure 2:**
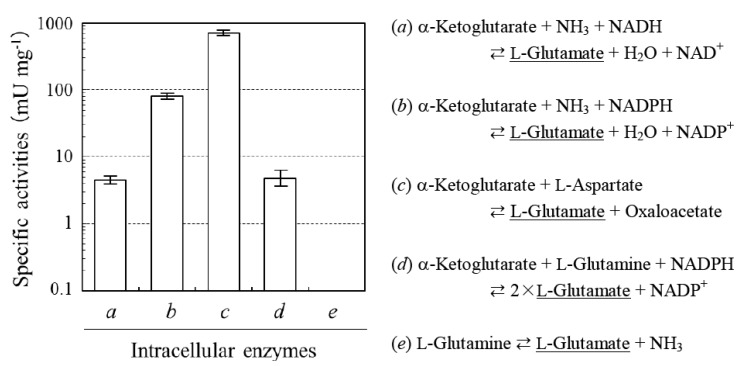
Intracellular activities for L-glutamate sources of *N. aegyptiaca *showing higher L-PGA productivity (means ± standard deviation; *n* = 5). One unit (U) was defined as the amount of enzyme that catalyzed the formation of 1 μmol of L-glutamate per min. Enzymes *a*, GDH (NADH); *b*, GDH (NADPH); *c*, GOT; *d*, GS; and *e*, GLS.

L-PGA is known to possess extremolyte-like applicability [6]. Extremolytes are exclusively isolated from extremophiles and protect biological macromolecules and cells from damage by external stress [7]. As L-glutamate serves as the best substrate for *in vivo *synthesis of microbial PGAs [4], we first assayed for the activities of enzymes involved in L-glutamate synthesis in *N. aegyptiaca*. Figure 2 revealed the presence of NAD(P)H-dependent L-glutamate dehydrogenases (GDH; *a* and *b*), pyridoxal 5’-phosphate-dependent L-aspartate aminotransferase (GOT; *c*), and NADPH-dependent L-glutamate synthase (GS; *d*). We were unable to detect the activity of NAD(P)^+^-dependent dehydrogenases using other amino acids (e.g., L-alanine, L-aspartate, L-lysine, L-phenylalanine, and L-serine) as a substrate. L-Glutaminase (GLS; *e*) was also inactive. *N. aegyptiaca* hence possesses multiple enzymatic sources of L-glutamate, similarly to well-characterized PGA producers from bacilli [4]. Moreover, the absence of glutamate oxidases indicated that glutamate anabolism predominates compared with its catabolism.

### 2.2. Absence of D-glutamate Suppliers in N. aegyptiaca

Although the pathways involved in D-glutamate metabolism have not, to our knowledge, been identified in *N. aegyptiaca*, the use of well-characterized PGA producers allowed us to conduct an in-depth investigation of the biosynthesis of D-glutamate [4]. Two distinct types of the suppliers have been confirmed [4] as follows: glutamate racemase (1) and D-amino acid aminotransferase (2).

L-Glutamate ⇄ D-glutamate (1)

D-Alanine + α-Ketoglutarate ⇄ Pyruvate + D-Glutamate (2)

In addition to these suppliers, we also examined the activity of an enzyme, NAD(P)H-dependent D-glutamate dehydrogenases (3).

α-Ketoglutarate + ΝΗ_3_ + NAD(P)H ⇄ D-Glutamate + Η_2_O + NAD(P)^+^ (3)

Under the conditions tested, neither glutamate racemase nor D-amino acid aminotransferase activity was detected. Moreover, our attempts to discover NAD(P)H-dependent D-glutamate dehydrogenases were unsuccessful, suggesting that the normal metabolism of *N. aegyptiaca* prevents the synthesis of D-glutamate and can eventually produce the L-homo type of PGA. This conclusion does not conflict with findings that PGA with different stereochemical conformations can be synthesized by the co-expression of a D-glutamate supplier, glutamate racemase, in an *Escherichia coli* strain genetically-engineered to produce PGA [8]. Besides, other amino acid racemases were absent in *N. aegyptiaca* as well, implying that this archaeon essentially has no potential for the production of D-amino acids.

**Figure 3:**
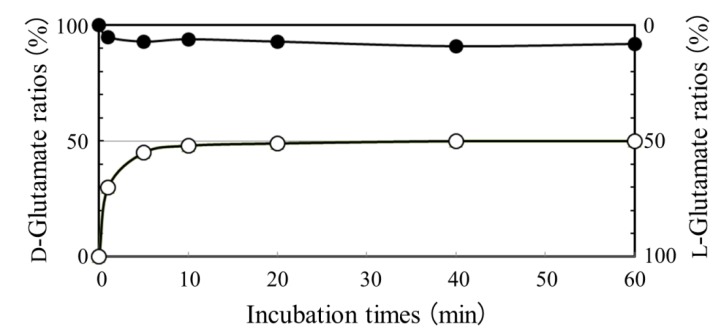
Time course of “L to D” (*open circles*) and “D to L” (*closed circles*) conversions in glutamate racemization of *B. subtilis*. If the D concentration is exceedingly higher than the L concentration in the coexistence of both enantiomers, the intracellular racemase may suffer a substrate inhibition due to its peculiar conformational change [9,10].

### 2.3. Analysis of a Pivotal D-glutamate Supplier in B. Subtilis Producing DL-PGA

*Bacillus subtilis *and *B. anthracis *produce DL- and D-PGA, respectively. The chirality of *B. subtilis* PGA is thus heterogeneous, whereas *B. anthracis *PGA possesses homochirality, which is symmetric to the PGA produced by *N. aegyptiaca*. In the present study, we focused our attention on amino acid racemase, a pivotal enzyme that can connect the L-amino acid world with its mirror world. In fact, only this racemase utilizes both enantiomers of amino acid substrates among known enzymes involved in amino acid biosynthesis. We thus examined the role of glutamate racemase in glutamate metabolism using the cytosolic fraction of *B. subtilis*. Figure 3 shows kinetics of the conversion (racemization) of glutamate from “L to D” and “D to L”. The data indicate that *B. subtilis *prefers the former conversion. Therefore, the glutamate racemase probably serves physiologically in the anabolism of PGA (to supply D-glutamate) rather than to catabolize PGA (degradation of the resulting D-glutamate) in a manner that contradicts published speculations [11,12].

## 3. Experimental Section

### 3.1. Microbes and Culture Conditions

*N. aegyptiaca *was cultured at 37 °C for 4 days in 200 mL of an S medium (pH 7.2) containing 25% NaCl, 0.2% KCl, 1% trisodium citrate, 0.75% casamino acids, and 1% yeast extract, and the cells were harvested by centrifugation. To enhance PGA productivity, harvested cells (0.4 g) were then cultured on a 2% agar plate (145 mm diameter) containing S medium (50 ml) at 37 °C for 2 weeks. Under these conditions, the productivity reached ~1.2 g/g of cells.

*B. subtilis* was cultured in 200 ml of Luria-Bertani medium [13] at 37 °C for 16 h and harvested by centrifugation. To induce PGA synthesis [14], harvested cells (0.4 g) were cultured at 37 °C for 4 days on a 1.5% agar plate (145 mm diameter) containing a GS medium (50 ml, pH 7.0) containing 2% L-glutamate, 5% sucrose, 1% (NH_4_)_2_SO_4_, 0.5% MgSO_4_•7H_2_O, 0.27% KH_2_PO_4_, 0.42% Na_2_HPO_4_, 0.05% NaCl, and 1% yeast extract. Under these conditions, PGA productivity reached ~0.8 g/g of cells.

### 3.2. Preparation of Cytosolic Fractions

The harvested cells (1 g) were suspended in 1 mL of a standard buffer (0.1 M Mops-NaOH (pH 7.0), 0.2 M KCl, and 5 mM dithiothreitol), sonicated (Branson, CT, USA), centrifuged at 12,000×G for 10 min, and further centrifuged at 39,000×G for 30 min. After dialysis at 4°C overnight against a 1,000-fold volume of the same buffer, all particulates were removed by filtration. The resulting supernatant (cytosolic fraction) does not show the activity of extracellular γ-glutamyltransferase, which liberates glutamate monomers from the N-terminal end of PGA [12], and was analyzed as described below. 

### 3.3. Enzyme Assays

#### 3.3.1. Sources of L-Glutamate

*Dehydrogenase*: The reaction mixture contained 0.1 M Tris-HCl (pH 8.0), 5 mM α-ketoglutarate, 0.4 mM NADH (or NADPH), and the cytosolic fraction (1 mg of proteins). Reactions were initiated by the addition of 80 mM NH_4_Cl and assayed by following NAD(P)H oxidation (decrease in absorbance at 340 nm or 360 nm) at 37 °C.

*Aminotransferase*: The reaction mixture contained 0.1 M Tris-HCl (pH 8.0), 50 mM L-aspartate, 0.1 mM pyridoxal 5’-phosphate, 0.4 mM NADH, commercially available malate dehydrogenase (MDH; 2.5 U), and the cytosolic fraction. Reactions were initiated by the addition of 10 mM α-ketoglutarate and assayed by coupling the reaction to MDH and following NADH oxidation (decrease in absorbance at 340 nm) at 37 °C.

*Synthase*: The reaction mixture contained 0.1 M Tris-HCl (pH 8.0), 5 mM α-ketoglutarate, 0.4 mM NADPH, and the cytosolic fraction. Reactions were initiated by the addition of 80 mM L-glutamine, and assayed by following NADPH oxidation (decrease in absorbance at 360 nm) at 30 °C.

*Glutaminase (Amidohydrolase)*: The reaction mixture contained 0.1 M Tris-HCl (pH 7.5), 5 mM NAD^+^, commercially available GDH (0.5 U), and the cytosolic fraction. Reactions were initiated by the addition of 50 mM L-glutamine and assayed by coupling the reaction to GDH and following NAD^+^ reduction (increase in absorbance at 340 nm) at 30 °C.

#### 3.3.2. D-Glutamate Suppliers

Reaction mixtures (0.2 mL) were incubated at 37 °C for indicated times, inactivated with 8 μLof 12 M HCl, neutralized with 16 μL of 6 M NaOH, and then diluted 5-fold with 2 mM CuSO_4. _A 5-μL sample was withdrawn and loaded onto a CHIRALPAK MA(+) column (4.6×50 mm; DAISEL, Tokyo, Japan) using an LC-10 HPLC system (Shimadzu, Kyoto, Japan). The column was eluted with 2 mM CuSO_4_ solution at the flow rate of 1 ml min^−1^, and the absorbance of the eluate was monitored at 235 nm. The elution volumes of D- and L-glutamate were approximately 10 and 15 ml, respectively. Yields (fmol) were estimated using the following equations: Y_D_ = 2.97× and Y_L_ = 2.91× (where × represents each peak area on the HPLC profiles) [15].

*Racemase*: The reaction mixture contained 0.1 M Tris-HCl (pH 8.0), 10 mM L-glutamate (or D-glutamate), and the cytosolic fraction.

*Aminotransferase*: The reaction mixture contained 0.1 M Tris-HCl (pH 8.0), 50 mM D-alanine, 10 mM α-ketoglutarate, 0.1 mM pyridoxal 5’-phosphate, and the cytosolic fraction.

*Dehydrogenase*: The reaction mixture consisted of the same components as the mixtures for the L-glutamate dehydrogenase assays described above.

#### 3.3.3. Other Enzymes

*Glutamate Oxidases* catalyze the following reaction: Glutamate + O_2_ + H_2_O → α-Ketoglutarate + NH_3_ + H_2_O_2_. These are two distinct types of glutamate catabolism catalyzed by L- or D-glutamate oxidases. The reaction mixture contained 0.1 M Tris-HCl (pH 8.0), 10 mM L- or D-glutamate, 0.03% 2,4-dinitrophenylhydrozine, and the cytosolic fraction. The formation of 2,4-dinitrophenylhydrozone of α-ketoglutarate after incubation at 37°C was followed by measuring the increase in absorbance at 550 mm. 

*Other amino acid racemases* catalyze the following reaction: L-amino acid(s) ⇄ D-amino acid(s). The reaction mixtures contained 0.1 M Tris-HCl (pH 8.0), 10 mM L-amino acid other than L-glutamate (e.g*.*, L-alanine, L-aspartate, L-lysine, L-phenylalanine, and L-serine), 0.1 mM pyridoxal 5’-phosphate, and the cytosolic fraction of *N. aegyptiaca*. After incubation at 37 °C, they were assayed by the HPLC methods [16]. 

### 3.4. Protein Assay

Protein concentrations were determined using a protein assay kit (Bio-Rad, CA, USA) using bovine serum albumin as a standard.

## 4. Concluding Remarks

From the perspective of polymer chemistry, a question arises whether homochiral biopolymers that possess highly complex structures that contribute to the selection and persistence of chirality were generated spontaneously in water, a solvent that randomizes the chirality of chemical compounds. We believe that it is necessary to discuss in detail the possibility that water could have participated in the chemical reactions that made life possible (Figure 1), and that the disruption of molecular chirality that characterizes biological processes in life may not have occurred in the environment before the birth of life. Archaea essentially inhabit an L-amino acid world owing to the absence of amino acid racemases, while eubacteria, to survive from adverse biological impacts, such as attack by proteases, envelop cell surfaces with peptidoglycans containing certain D-amino acids.

**Figure 4:**
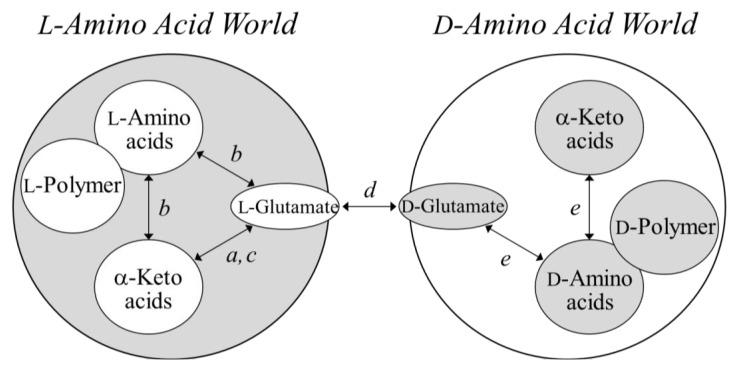
L-Glutamate-dominant world, its mirror world, and a pivotal enzyme connecting both worlds. Enzymes *a*, GDHs; *b*, L-amino acid aminotransferases including GOT; *c*, GS; *d*, glutamate racemase; and *e*, D-amino acid aminotransferase.

Mammals were once believed to reside in an L-amino acid world along with archaea; however, D-serine has been identified in humans and other animals as a mediator of memory, synthesized by the enzyme “serine racemase” [17]. Although D-PGA is not toxic to mammals, it nullifies the immunity of hosts and can contribute to the severity of diseases “anthrax” [4]. These may suggest a novel story based on the view that “no chirality–breaking, no evolution (or no adaptation)” for life based on homochirality. If so, when did the first glutamate racemase appear in the L-amino acid world? Can a mirror form of glutamate racemase, created only from D-amino acids, yet catalyze the racemization of glutamate? It may be the case that the glutamate racemase will be considered as the pivotal enzyme responsible for modifying the present L-dominant glutamate-metabolic pathways in nature (Figure 4).
